# Consistent opening of the blood brain barrier using focused ultrasound with constant intravenous infusion of microbubble agent

**DOI:** 10.1038/s41598-020-73312-9

**Published:** 2020-10-06

**Authors:** Norman A. Lapin, Kirt Gill, Bhavya R. Shah, Rajiv Chopra

**Affiliations:** 1 Focused Ultrasound Laboratory, Department of Radiology, UT Southwestern Medical Center, Dallas, TX 75390 USA; 2 Department of Neurosurgery, UT Southwestern Medical Center, Dallas, TX 75390 USA; 3 Peter O’Donnell Jr. Brain Institute, UT Southwestern Medical Center, Dallas, TX 75390 USA; 4 Advanced Imaging Research Center, UT Southwestern Medical Center, Dallas, TX 75390 USA

**Keywords:** Preclinical research, Medical research, Neuroscience, Blood-brain barrier, Techniques and instrumentation, Characterization and analytical techniques, Magnetic resonance imaging

## Abstract

The blood brain barrier (BBB) is a major obstacle to the delivery of therapeutics to the brain. Focused ultrasound (FUS) in combination with microbubbles can non-invasively open the BBB in a targeted manner. Bolus intravenous injections of microbubbles are standard practice, but dynamic influx and clearance mechanisms prevent delivery of a uniform dose with time. When multiple targets are selected for sonication in a single treatment, uniform serum concentrations of microbubbles are important for consistent BBB opening. Herein, we show that bubble infusions were able to achieve consistent BBB opening at multiple target sites. FUS exposures were conducted with different Definity microbubble concentrations at various acoustic pressures. To quantify the effects of infusion on BBB opening, we calculated the MRI contrast enhancement rate. When infusions were performed at rates of 7.2 µl microbubbles/kg/min or below, we were able to obtain consistent BBB opening without injury at all pressures. However, when infusion rates exceeded 20 µl/kg/min, signs of injury occurred at pressures from 0.39 to 0.56 MPa. When compared to bolus injections, a bubble infusion offers a more controlled and consistent approach to multi-target BBB disruption.

## Introduction

The blood brain barrier (BBB) is composed of a continuous layer of endothelial cells connected by tight junctions and surrounded by pericytes, non-fenestrated basal laminae, and astrocyte foot processes that separate the systemic circulation from CNS tissues^1–3^. The BBB serves as a critical regulator of homeostasis in the central nervous system (CNS) and is the first line of defence against toxins and microorganisms. Predictably, the BBB is also a significant hurdle to the delivery of drugs and therapeutics to the brain. Only lipid soluble molecules smaller than 400–500 Da can naturally cross the BBB. These conditions exclude 98% of drugs^4^.

Historically, methods to bypass the BBB have included intra-arterial hyperosmotic solutions which cause endothelial cell shrinkage and vasodilation leading to paracellular transport via separation of tight junctions^5,6^. Additionally, attempts have been made to target cell surface receptors, but these methods are limited by targeting affinity^2,7^, systemic clearance, and uptake by the mononuclear phagocytic system^8,9^. Alternatively, intrathecal and/or intraventricular methods have been used to bypass the BBB. However, these methods are invasive and limited by the blood-CSF barrier. In addition to the limitations mentioned above, these methods lack the ability to target precise regions of the brain.

A method that overcomes these limitations is focused ultrasound (FUS) in the presence of systemically circulating microbubbles. Transcranial FUS results in oscillations of intravascular microbubbles^10^ that stimulate the opening of the BBB^11,12^. FUS has been explored as a method to deliver therapies across the BBB for malignancies^13^, neurodegenerative diseases^14,15^, and movement disorders^15–17^. The technology has also been shown to be safe and efficacious in patients with Alzheimer’s Disease and brain tumors^18,19^.

The safety and reliability of BBB opening with FUS depends on a number of parameters: acoustic pressure amplitude^20^, burst length^21^, and pulse repetition frequency^22^, among others. Studies have shown that these and other parameters affect the acoustic emissions of microbubbles. FUS systems with acoustic feedback capabilities have been able to characterize these acoustic emissions and apply them to real-time feedback control^13,23–28^. Feedback control enables the user to dynamically adjust FUS system parameters to prevent damage and ensure reproducible BBB opening^18,29^.

Consistent BBB opening is also highly dependent on the type and size of microbubble selected, microbubble dose, and how the microbubbles are delivered^18^. In the majority of FUS protocols, microbubbles are administered via bolus intravenous injection. As a result, systemic bubble dose is dynamic, rising and falling within minutes^10,23,27,30^. Variability in bubble concentrations can prevent safe and consistent BBB opening and pose a formidable challenge for reproducible, real-time acoustic feedback control^18^. Furthermore, the clearance of microbubbles limits the time during which BBB opening can be performed. Multiple bolus injections are required when more than one target is selected. Microbubble infusions address these limitations by maintaining an acceptable serum bubble concentration for the duration of the treatment.

In the present study, we demonstrate that a microbubble infusion is able to consistently open the BBB in sequentially targeted FUS points during a single treatment. We evaluated our bubble infusion protocol by varying the microbubble concentration, acoustic pressure, and duration of the infusion when disrupting the BBB in pre-determined targets. We quantified our results by calculating the enhancement rate of T1-weighted MR images post injection of contrast agent, routinely obtained to confirm BBB disruption. We also compared the effectiveness of BBB disruption using our infusion protocol verses bolus injection. We further obtained T2-weighted images to determine injury thresholds.

## Methods

### Microbubbles

Definity (Perflutren Lipid Microsphere) ultrasound contrast agent was purchased from Lantheus Medical Imaging (N. Billerica, MA, USA). The mean bubble diameter is given by the manufacturer as ranging from 1.1 to 3.3 µm, therefore the centre of this range, 2.2 µm, was used for bubble gas volume calculations. Based on supplier specifications, a single vial contains up to 1.2 × 10^10^ perflutren lipid microspheres once activated. As such we approximated 1 × 10^10^ microbubbles/1500 µl bubble agent/vial for all calculations.

### FUS system

**Figure 1:**
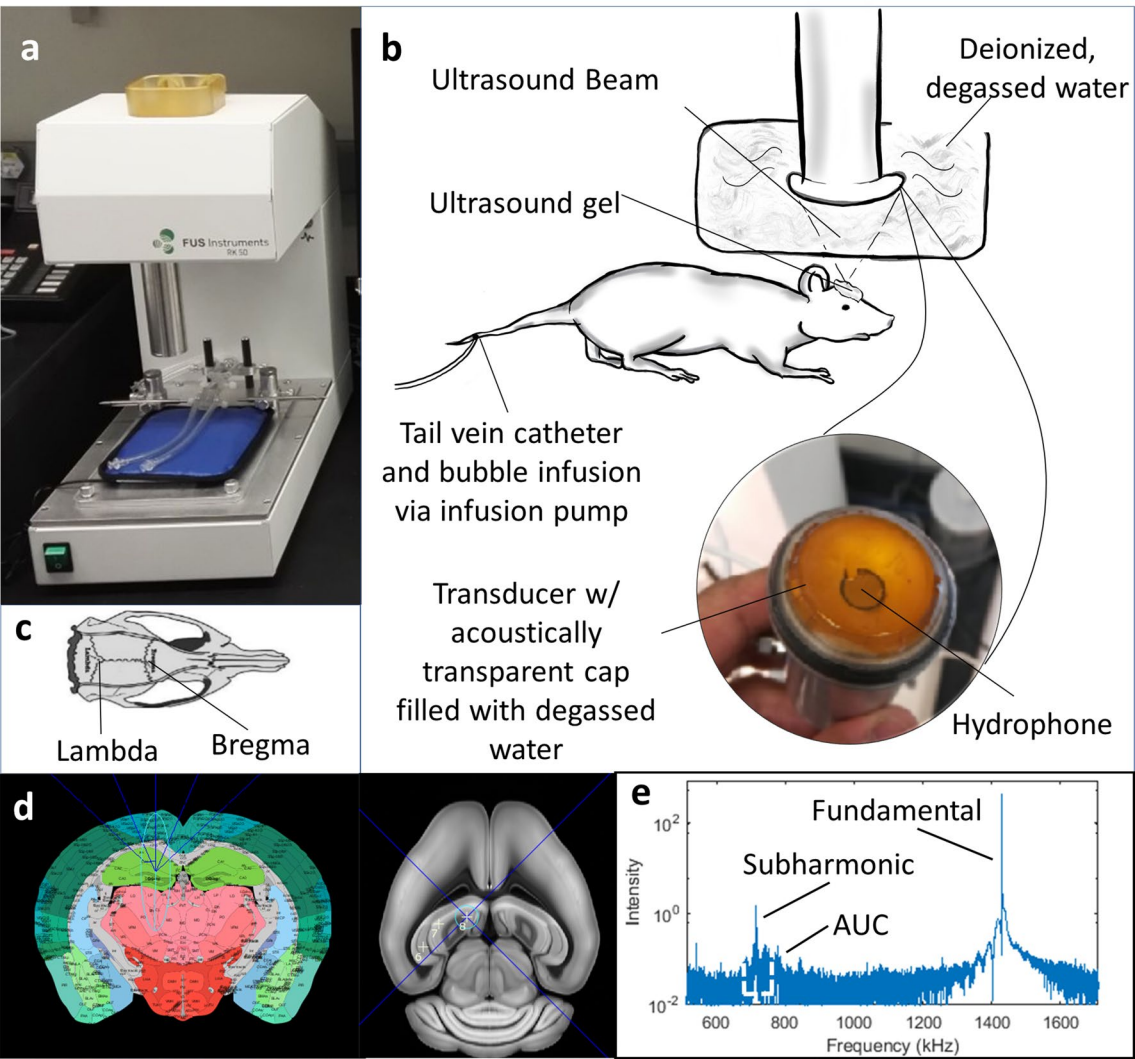
Representation of FUS procedure, system, and software. (**a**) The RK50 stereotactic system from FUS Instruments used during our experiments. (**b**) A schematic of the experimental set-up used in this study. Of note, a 27-gauge tail vein catheter was used during our procedure for delivery of the bubble infusion. (**c**) Skull landmarks lambda and bregma are identified with the stereotactic system after an incision has been made in the mouse’s shaved scalp. (**d**) A Python-based software uses a mouse brain atlas registered to the identified skull landmarks to carry out a sonication treatment plan of multiple sequential points. (**e**) A hydrophone returns an acoustic signal that is transformed by the software into a visual representation of the acoustic spectra from which the area under the curve (AUC) is calculated. The authors thank April Gill for creating the drawing in (**b**) and kindly providing it to us for publication.

The FUS system (Fig. 1a) is a stereotactic-guided system (RK-50, FUS Instruments, Toronto, ON, CA) utilizing landmark registration (Fig. 1c) to a mouse atlas (Fig. 1d) for localization of targets. Target coordinates and FUS parameters were entered into the system software to form a treatment plan (Fig. 1d). A 1.43 MHz ultrasound transducer with a 35 mm diameter and 24.5 mm radius of curvature executed the ultrasound emissions (Fig. 1b). The pressure output and beam profile of the transducer were calibrated with a needle hydrophone (Precision Acoustics, UK). A hydrophone concentric with the transducer (Fig. 1b) returns an acoustic signal of the infused bubbles that provides acoustic spectra from which quantitative data can be obtained as the area under the curve (AUC) of the subharmonic spectral peak (Fig. 1e).

### Animal preparation

Swiss Webster mice (Charles River, Wilmington, MA, USA) of both genders, 3 to 4 months of age were utilized. Each animal was anesthetized through inhalation of 2–3% isoflurane and 1–2 L/min of 100% oxygen or medical air. A 27-gauge I.V. catheter assembly with a dead volume of 69 µl was placed in the tail vein for bubble administration and affixed with tissue glue. A physiologic monitoring system (PhysioSuite, Kent Scientific Corp., Torrington, CT, USA) was used to monitor respiration and rectal temperature throughout the experiment, while a heating pad under the animal was used to maintain body temperature. Hair over the cranial surface of the skull was removed using an animal clipper and depilatory cream (VEET sensitive formula, Reckitt Benckiser, Parsippany, NJ, USA). Using sterile technique, a 1–2 cm incision was performed over the skull to visualize lambda and bregma skull suture landmarks (Fig. 1c). The animal was stabilized on the stereotaxic apparatus using ear bars and a bite bar (Fig. 1b). A concentration of 1–3% isoflurane was delivered with medical air to maintain anaesthesia during ultrasound exposures. Once the skull landmarks were established, ~ 0.5 ml ultrasound gel was added to the animal’s skull for acoustic coupling, taking care to avoid air bubbles. A tank filled with deionized and degassed water was lowered onto the ultrasound gel to a fixed point coincidental with the skull, and the transducer was lowered into the tank ready to begin sonication (Fig. 1b).

### Bubble preparation and delivery

We varied the bubble dose to span prior clinical and pre-clinical studies. The manufacturer recommendations for infusion administration are dilution of 26 µl of Definity per ml of preservative-free saline to be delivered at a rate of 4–10 ml/min. The manufacturer recommendations for bolus administration, are a dose of 10 µl/kg delivered over 30–60 s. Clinical studies have utilized bolus delivery at a dosage of 4–20 µl/kg^29^, while bolus dosages in preclinical studies have ranged from 4.3 to 1000 µl/kg^14,31,32^. The microbubble doses used in the present study ranged from 10 to 700 µl/kg. Infused microbubble dose was expressed in terms of influx of microbubble volume per animal weight per time infused (µl/kg/min) to normalize for delivery rates across experiments.

### FUS exposures

The FUS transducer was submerged in degassed and deionized water. Then an acoustically transparent cap was attached to trap water between the cap and the concave surface of the transducer. A 30 s ultrasound exposures was delivered to the hippocampus and frontal cortex targets. Each 30 s exposure consisted of 30 bursts with a pulse length of 10 ms and a repetition period of 1000 ms. The non-derated focal pressure was between 0.39 and 0.56 MPa.

### Infusion experiments

**Figure 2:**
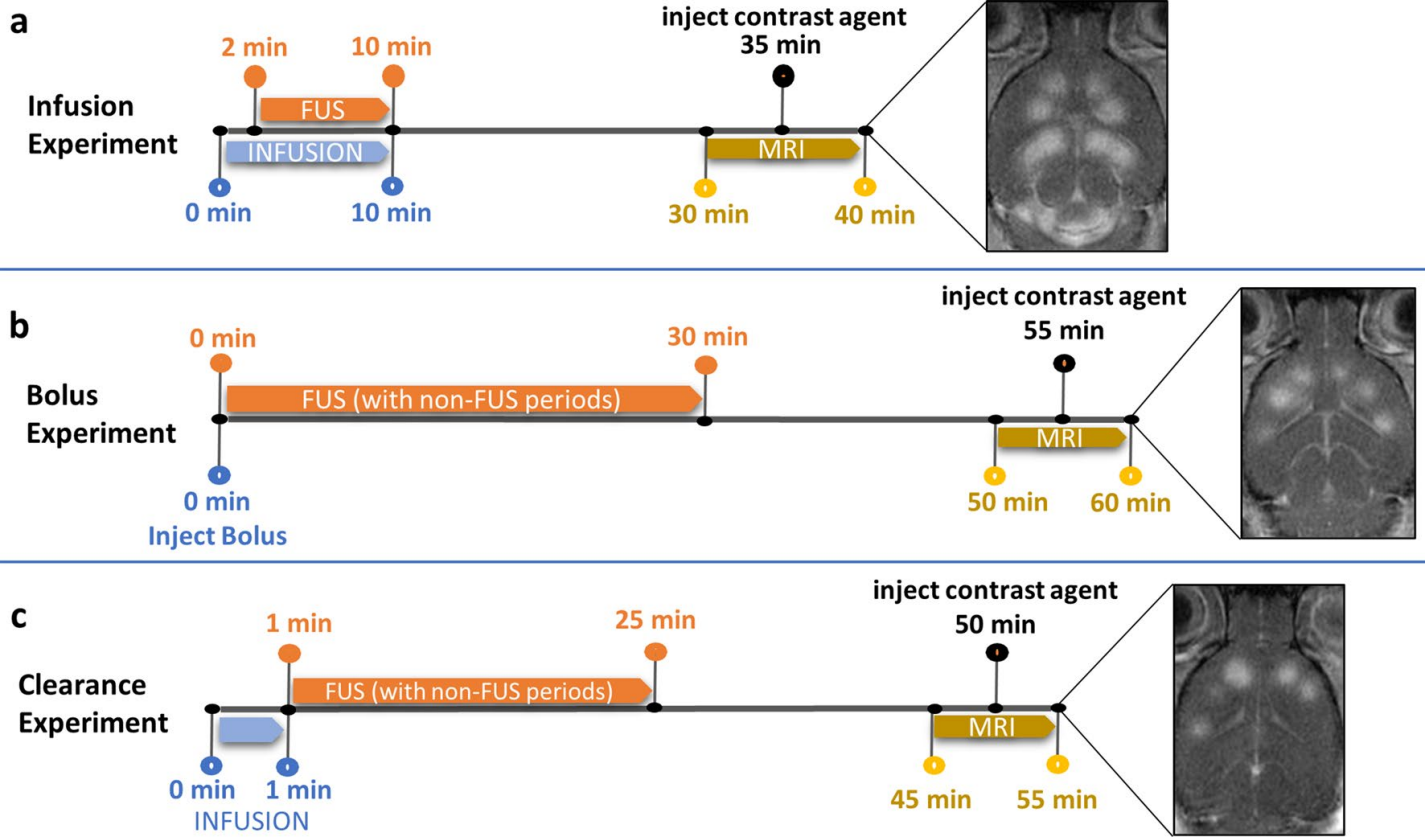
Experimental timelines for standard infusion, bolus and microbubble clearance experiments conducted in this study. (**a**) A representative timeline of a standard infusion experiment, where the infusion is started prior to FUS to allow for an initial build-up of bubble volume in circulation and is continued throughout FUS in the treatment of sequential points. (**b**) In contrast, a representative timeline of a bolus experiment demonstrates the immediate start of FUS after bolus injection. During sonication, non-treatment periods were included to observe the effects of bubble clearance. (**c**) To further elucidate the differences in bubble clearance between bolus and infusion experiments, a clearance experiment protocol simulates a bolus injection with a pre-infusion period and the stoppage of infusion at the onset of FUS. Again, the inclusion of non-treatment periods within the treatment plan prolongs FUS, allowing for bubble clearance. All three experiment protocols finish with T2 and T1 pre and post contrast MR image collection.

Definity microbubble agent was activated, diluted in saline, and transferred to a 1 ml syringe, which was then placed in an infusion pump (Nanojet, Chemyx Inc, Stafford, TX, USA). A catheter (PE-20 tubing) was used to connect the syringe to the tail vein. The infusion volumetric flow rate for all experiments was 50 µl/min, while the microbubble infusion dosage rate ranged from 1.74 to 93.8 µl/kg/min. Ultrasound exposures were initiated after infusing a volume of 69 µl to account for the dead space of the tail vein catheter (Fig. 2a). The sonication of each point took approximately 45 s to complete, including a few seconds for the FUS transducer to move from one target to the next. After completion of the treatment session, actual sonication times for each target were obtained from the software log files.

### Bolus experiments

Definity microbubble agent was activated, diluted in saline, transferred to a 1 ml syringe and injected into a tail vein catheter (Fig. 2b). Dilutions were calculated to achieve approximate dosages of 100 and 200 µl/kg, typical of bolus experiments in the literature^9,14,22^. FUS was commenced immediately after bolus injection. Sonication targets alternated between the right and left hemispheres to mitigate location-based bias. Two-minute delays were introduced between sonications to observe the effects of bubble clearance both during and beyond the expected time for bubbles to clear circulation. Actual sonication times for each target were obtained as specified in infusion experiments.

### Microbubble clearance experiments

Microbubbles were activated, diluted and setup in the infusion pump according to the same procedure as in infusion experiments. Dilutions were calculated to achieve dosages similar to those used in the bolus experiments by selecting microbubble concentrations that would reach those systemic dosages after several seconds of infusion. Microbubble infusion was stopped immediately after sonication of the first point was completed, marking the start of systemic microbubble clearance unobscured by simultaneous infusion (Fig. 2c). The enhancement rate of the first treated point was taken as a positive control to which all subsequent points were referenced to determine percent decline in enhancement rate during clearance. Similar to the bolus experiment protocol, the sequential order of sonicated spatial locations was alternated between brain hemispheres, and 2-min delays were inserted between each successive sonication. Actual sonication times for each target were obtained as specified in infusion experiments.

### MR image acquisition

After sonication was complete, BBB opening was confirmed with MR imaging. The animal was placed in a custom-made animal bed and kept anesthetized with 2–3% isoflurane at 1 L/min flow of oxygen. The bed was placed in a small animal 7.0 T (16-cm horizontal bore) magnetic resonance scanner (Varian, Palo Alto, CA) with a 38 mm volume RF coil run on ParaVision software version 6.0.1 (Bruker BioSpin MRI GmbH). A small animal monitoring system (Small Animal Instruments, Inc., Stony Brook, NY) with temperature-controlled feedback measured animal respiration and controlled body temperature using heated air blown through the bore of the magnet throughout the imaging session. Rectal temperature was maintained at 35.5 ± 1.0 °C.

Gradient echo T1-weighted MR images were acquired through a fast low-angle shot sequence (echo time (TE) = 2.76 ms, repetition time (TR) = 106.65 ms, flip angle 20°, field of view (FOV) = 256 × 256 mm, slice thickness = 1.0 mm) before and after injection of contrast agent, Gadobutrol (Gadovist, Bayer Healthcare Pharmaceuticals Inc, Whippany, NJ, USA) administered via tail catheter (1.0 mmol/kg) followed by a 70 µL saline flush. T2-weighted images were acquired through a spin-echo TurboRARE sequence (TE = 40 ms, TR = 2500 ms, flip angle 90°, Rare factor 8, 6 averages, FOV = 256 × 256 mm, slice thickness = 1.0 mm) prior to contrast agent injection (to avoid obfuscation of image features). All images were acquired in the axial anatomical plane to capture lateral cross-sections in the focal zone of the FUS beam where BBB opening was targeted.

### Data collection and processing

#### Assessment of BBB opening via T1-weighted contrast enhancement

Using the DICOM software Horos (HorosProject.org), circular regions of interest (ROIs) of equal area were drawn within FUS treated regions on the two or three centremost slices of MR T1-weighted scans both before (precontrast) and after (postcontrast) injection of contrast agent for each animal (Fig. 2a–c). Of the two to three ROIs measured on different slices at a particular target point, the ROI of greatest mean intensity in the postcontrast image was taken to be closest to the lateral cross-section of the focal zone in the FUS beam. The mean intensity of this ROI and that of the corresponding precontrast ROI were used to calculate enhancement rate according to Eq. (1).

1 $${\text{Enhancement rate}} = \frac{{{\text{Postcontrast intensity}} - {\text{Precontrast intensity}}}}{{\text{Precontrast intensity}}}$$

The standard deviation of each enhancement rate for each ROI was calculated according to error propagation of the standard deviations of corresponding precontrast and postcontrast intensities. Enhancement rate error propagation is expressed in Eq. (2) where pre = precontrast mean intensity, post = postcontrast mean intensity, Δpre = standard deviation of precontrast intensity, and Δpost = standard deviation of postcontrast intensity.

2 $${\text{Standard deviation of enhancement rate}} = \left| {\text{Enhancement rate}} \right| \times \sqrt {\frac{{\Delta {\text{post}}^{2} + \Delta {\text{pre}}^{2} }}{{\left( {{\text{post}} - {\text{pre}}} \right)^{2} }} + \left( {\frac{{\Delta {\text{pre}}}}{{{\text{pre}}}}} \right)^{2} }$$

#### Assessment of tissue damage on T2-weighted images

FUS target sites were assessed qualitatively (presence versus absence of T2 changes) on T2-weighted images for hypointense signal suggestive of haemorrhage or oedema.

### Data analysis

Statistical analysis of enhancement rates between brain hemispheres treated at different acoustic pressures was performed using an online unpaired *t* test^33^. For binary data (the presence or absence of post-FUS T2 changes suggestive of tissue damage at different microbubble doses) an online two-tailed two population proportion test was used^34^.

### Image processing

Window level (WL) and window width (WW) of all contrast-enhanced T1-weighted images were adjusted using RadiAnt DICOM Viewer software (version 2020.1.1, Medixant, Poznań Poland) such that brain tissue across animals scaled at approximately the same brightness and contrast. To compensate for differences in image brightness and contrast due to variability in contrast agent dose between imaging sessions, WL was set to ~ 2× the intensity of untreated brain tissue proximal to the location of treated points, and WW was set to ~ 2× WL. Image adjustments were made solely for presentation purposes and did not affect ROI values.

Similar adjustments were performed on T2-weighted images such that changes suggestive of tissue damage were made clearly visible. Here, the intensity of tissue external and proximal to brain parenchyma was subtracted from ~ 3× the mean intensity of untreated brain tissue, and WW was set to this difference. WL was then set to 2/3 of WW.

### Ethics statement

All experiments were performed with the approval of the Institutional Animal Care and Use Committee of the University of Texas Southwestern Medical Center. All methods and protocols were carried out in accordance with the guidelines set forth in the Guide for the Care and Use of Laboratory Animals.

## Results

**Figure 3:**
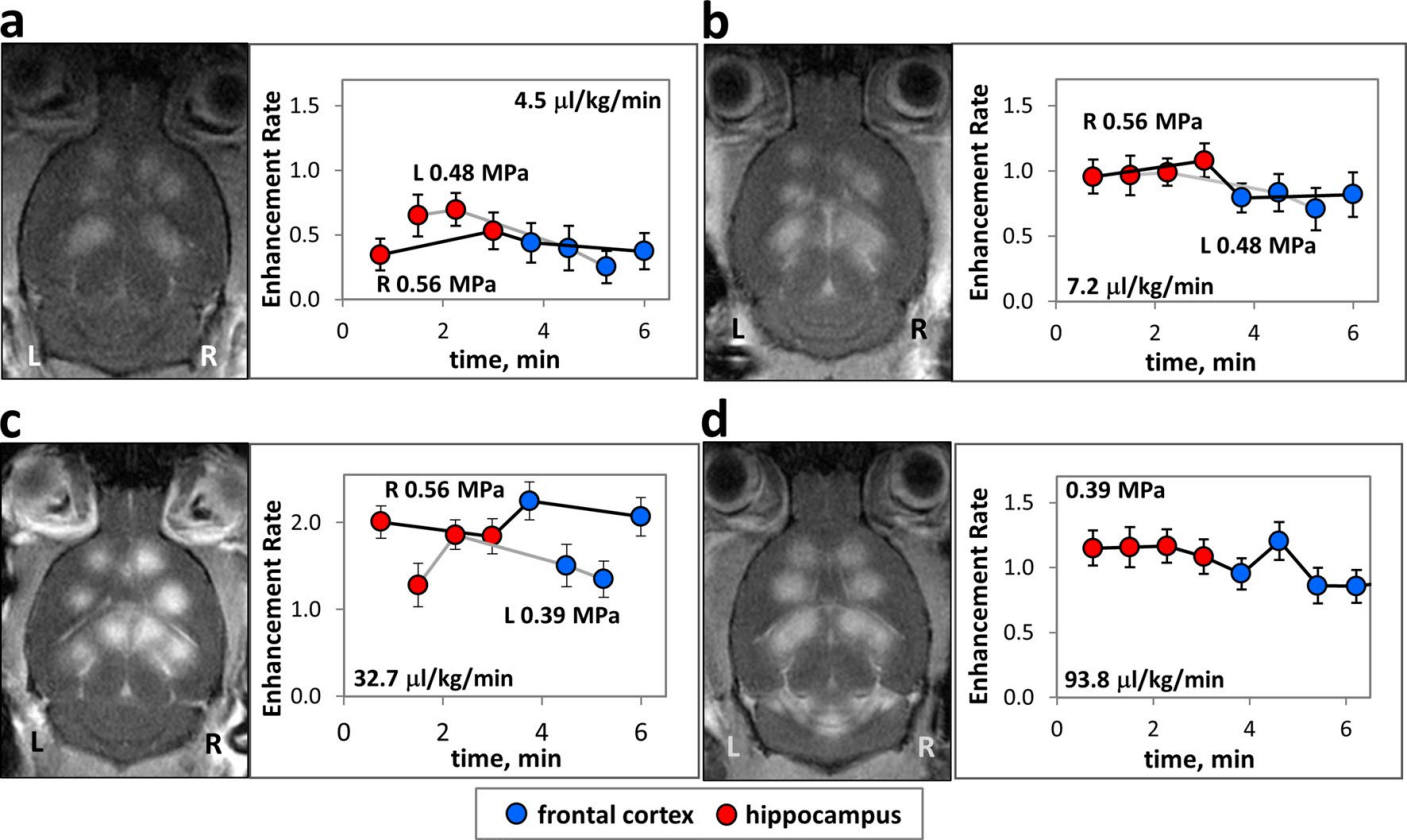
Enhancement rate versus time under constant infusion of microbubbles during FUS treatment. Representative MR images and corresponding plots of the brains of treated animals post injection of contrast agent showing FUS treated points. Enhancement rate vs. time post commencement of FUS treatment for (**a**) 4.5 (**b**) 7.2 (**c**) 32.7 and (**d**) 93.8 μl Definity microbubble agent/kg animal/min infused, a measure of microbubble dosage influx into animal circulation. In (**a**–**c**) each hemisphere of the brain (R, right and L, left) is treated at a specified acoustic pressure identified by connection of points with either black (higher pressure) or grey (lower pressure) lines. An animal treated at a single acoustic pressure in both hemispheres is shown in (**d**). WL and WW of images have been adjusted as stated in “Methods” section.

To understand the relationship between microbubble infusion dosage rates, acoustic pressures, and BBB opening, we sonicated different regions of the brain under variable conditions and then calculated enhancement rates. As expected, for a given animal at constant bubble influx and acoustic pressure, enhancement rates remained relatively consistent from point to point over time and across parenchymal regions (Fig. 3).

### Infusion experiments

The purpose of these experiments was to quantify the temporal dependence of BBB opening with infusion of microbubbles during FUS across different regions of the brain in a single treatment session. To study the effect of acoustic pressure on BBB opening with different infusion rates, the regions of the brain targeted were kept constant while the acoustic pressures varied between one side and the other. At constant dosage rates of 1.7–7.2 µl/kg/min, the left hemisphere was treated with an acoustic pressure of 0.48 MPa and while on the right, the acoustic pressure was increased to 0.56 MPa (Representative animals, Fig. 3a, b). Despite the increase in acoustic pressure, there was no significant difference in mean enhancement rates between sides. However, at an infusion rate of 32.7 µl/kg/min, and acoustic pressure increase from 0.39 MPa on the left side to 0.56 MPa on the right, there was a clear increase in enhancement rates from 1.50 ± 0.26, left to 2.04 ± 0.17, right, significant at *p* = 0.013 for n = 4 points per side (Fig. 3c). When acoustic pressure was held constant at 0.39 MPa between left and right sides at the highest dosage rate of 93.8 µl/kg/min (Fig. 3d), the point to point variability in enhancement rates (standard deviation of 0.14 across mean point values) was similar to that at low dosage rates (standard deviations from 0.08 to 0.21, Fig. 3a,b), even though acoustic pressures varied between hemispheres. See Supplementary Table S1 online.

**Figure 4:**
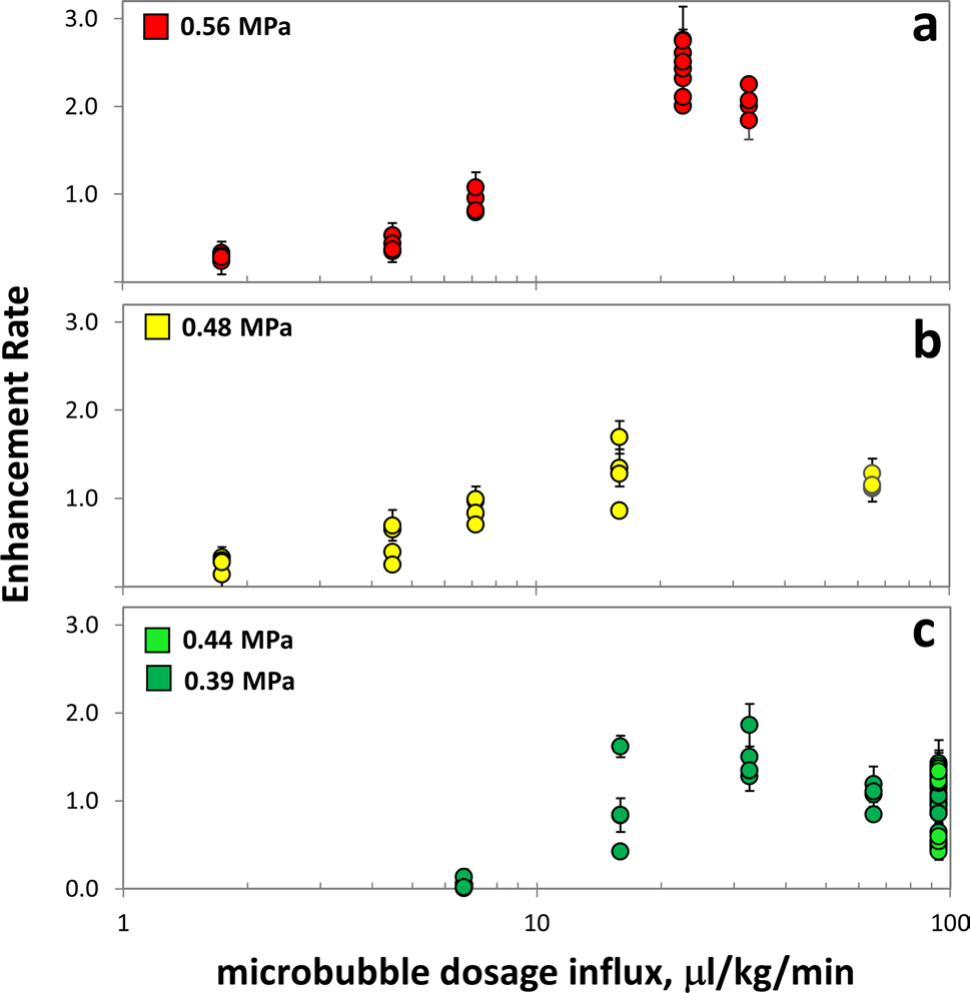
Enhancement rate versus microbubble dosage rate infused into animal circulation during FUS treatment of consecutive points in the brain at different acoustic pressures across animals. Enhancement rates are shown for microbubble infusions performed at acoustic pressures of (**a**) 0.56 MPa (**b**) 0.48 MPa and (**c**) 0.39 and 0.44 MPa. For each treated point, error bars express enhancement rate standard deviations that have been propagated from standard deviations of pre-contrast and post-contrast ROIs.

At 0.39 MPa and a dosage rate of 7.2 µl/kg/min, enhancement rates were close to background levels (Fig. 4c). When the dosage rate was raised to 15.9 µl/kg/min, the mean enhancement rate for the treated points rose to 0.93 ± 0.50. At 32.7 µl/kg/min, enhancement rates rose further to 1.50 ± 0.26. However, with greater increase in microbubble dosage rate to 65.2 µl/kg/min, enhancement rates unexpectedly decreased to 1.05 ± 0.15 (*p* = 0.024, compared to enhancement rates at 32.7 µl/kg/min, n = 4 both groups). At the highest bubble dosage rates tested (four animals at 93.8 µl/kg/min), mean enhancement rates ranged from 0.53 ± 0.6 to 1.22 ± 0.10 with substantial variation in BBB opening between animals.

At increased acoustic pressures of 0.48–0.56 MPa (Fig. 4a,b), we observed enhancement rate fluctuations similar to those observed at 0.39 MPa. At the lowest microbubble dosage rate tested (1.7 µl/kg/min), mean enhancement rates were 0.26 ± 0.08 at 0.48 MPa and 0.29 ± 0.04 at 0.56 MPa. As microbubble dosage rate increased, enhancement rates rose concomitantly reaching 1.29 ± 0.34 at 0.48 MPa and 15.9 µl/kg/min and as high as 2.44 ± 0.28 at 0.56 MPa and 22.7 µl/kg/min. Then, similar to behaviour at lower pressure, at 0.48 MPa and 65 µl/kg/min, enhancement rates dropped to 1.17 ± 0.08 (not significant). At 0.56 MPa and 32.7 µl/kg/min, enhancement rates decreased to 2.04 ± 0.17 (*p* = 0.027 with n = 4, compared to enhancement rate at 22.7 µl/kg/min with n = 8).

### Bolus experiments

**Figure 5:**
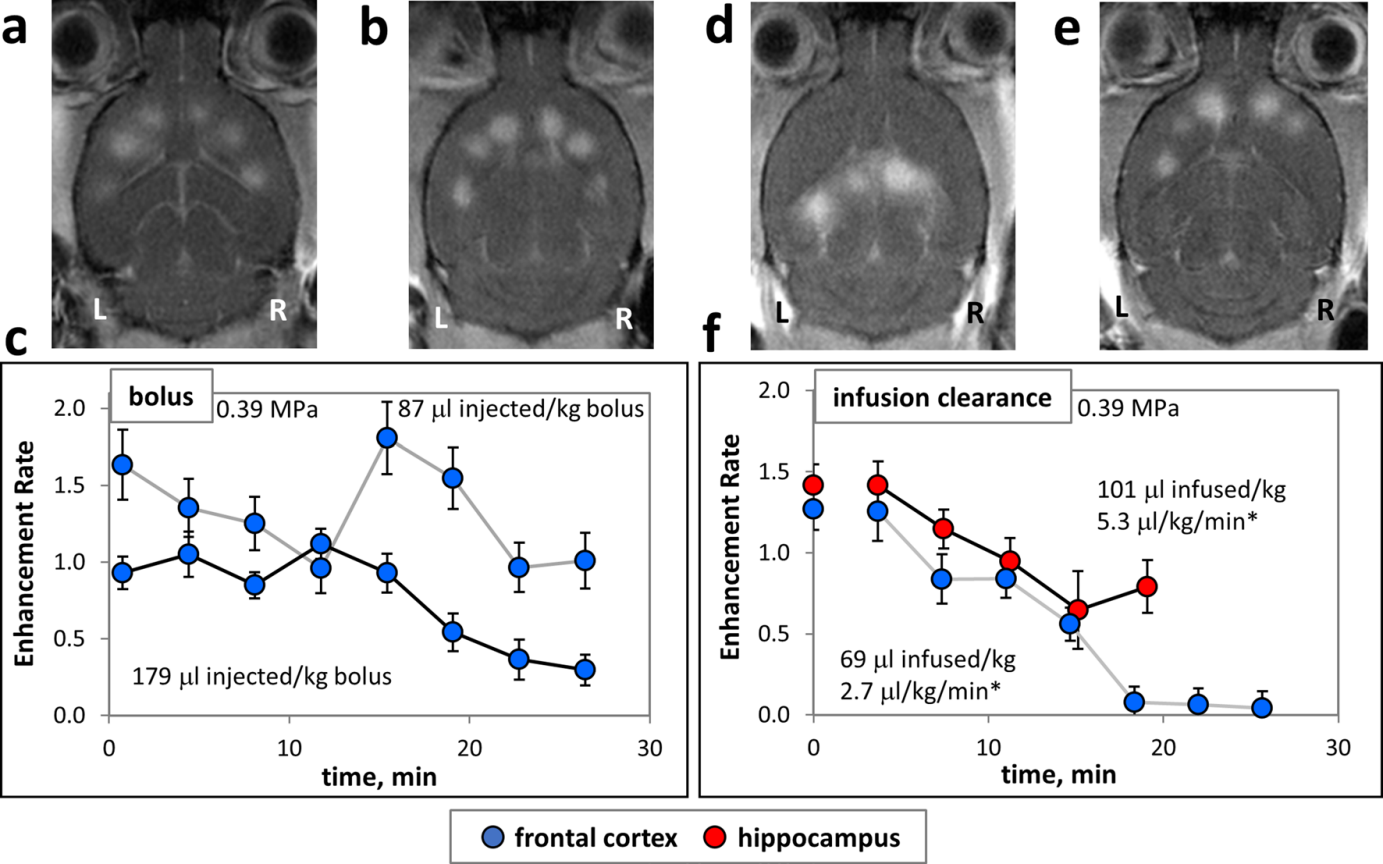
Enhancement rate dynamics with FUS treatment during microbubble systemic clearance. (**a**–**c**) Systemic bolus injections of microbubble solutions with FUS treatment commencing immediately after injection. Representative MR images of FUS treated brains at points in the frontal cortex post (**a**) 3.3 µl/kg/min bolus and (**b**) 6.8 μl/kg/min bolus injections. (**c**) Plots of enhancement rate versus time post start of FUS treatment corresponding to images in (**a**,**b**). (**d**–**f**) Infusion clearance experiments where microbubbles were infused briefly and FUS treatment began immediately after infusion was stopped. Representative MR images of FUS treated brains at points in (**d**) hippocampus and (**e**) frontal cortex. (**f**) Plots of enhancement rate versus time after the start of FUS treatment corresponding to images in (**d**,**e**). *Average microbubble dosage rates are calculated as total volume of microbubble agent delivered over total treatment time. Points treated in frontal cortex are shown in blue, and rostral hippocampus in red. For each animal (represented by each trace in the plots) the total quantity of microbubbles delivered (in μl of equivalent undiluted agent) per kg is given. Dividing by total duration of FUS treatment yields a nominal bubble agent dose delivery per treatment time, permitting comparison to other experiments in this study. WL and WW of images have been adjusted as stated in “Methods” section.

The purpose of these experiments was to quantify the temporal dependence of BBB opening with bolus injections. In our sample bolus experiments (Fig. 5a–c), enhancement rate persistence was surprisingly longer than expected. At 12 min following an 87 µl/kg bolus injection, enhancement rates reached a minimum of 59% of the initial baseline, after which they fluctuated for the remainder of the 26-min treatment. Enhancement rates of a larger 179 µl/kg bolus in a second animal did not substantially diminish until more than 15 min after injection, reaching a minimum of 18% of the initial value at the last treatment point at 26 min. Mean enhancement rates across points in each treatment were 1.32 and 0.76 for small and large boluses, respectively, with corresponding standard deviations across mean point values of 0.33 and 0.32. These standard deviations were substantially greater than most in the infusion experiments (see Supplementary Table S1 online).

### Bubble infusion clearance experiments

The purpose of these experiments was to quantify the temporal dependence of BBB opening during systemic bubble clearance. In these sample clearance experiments (Fig. 5d–f), microbubble doses of 101 µl/kg and 69 µl/kg were infused, and after infusion was stopped enhancement rates demonstrated initial expected decline (Fig. 5f). However, against our expectations, 15 min passed after cutting off infusion before the enhancement rate of the 101 µl/kg dose reached a minimum of 46% of its baseline value. For the 69 µl/kg dose, 18 min passed before it reached 6% of its baseline value. In contrast to the time-dependent changes in enhancement rate in the bolus experiments, enhancement rates during bubble clearance were substantially more uniform in their decline over time.

### Evaluation of T2 heterogeneity at various bubble dosage rates

**Figure 6:**
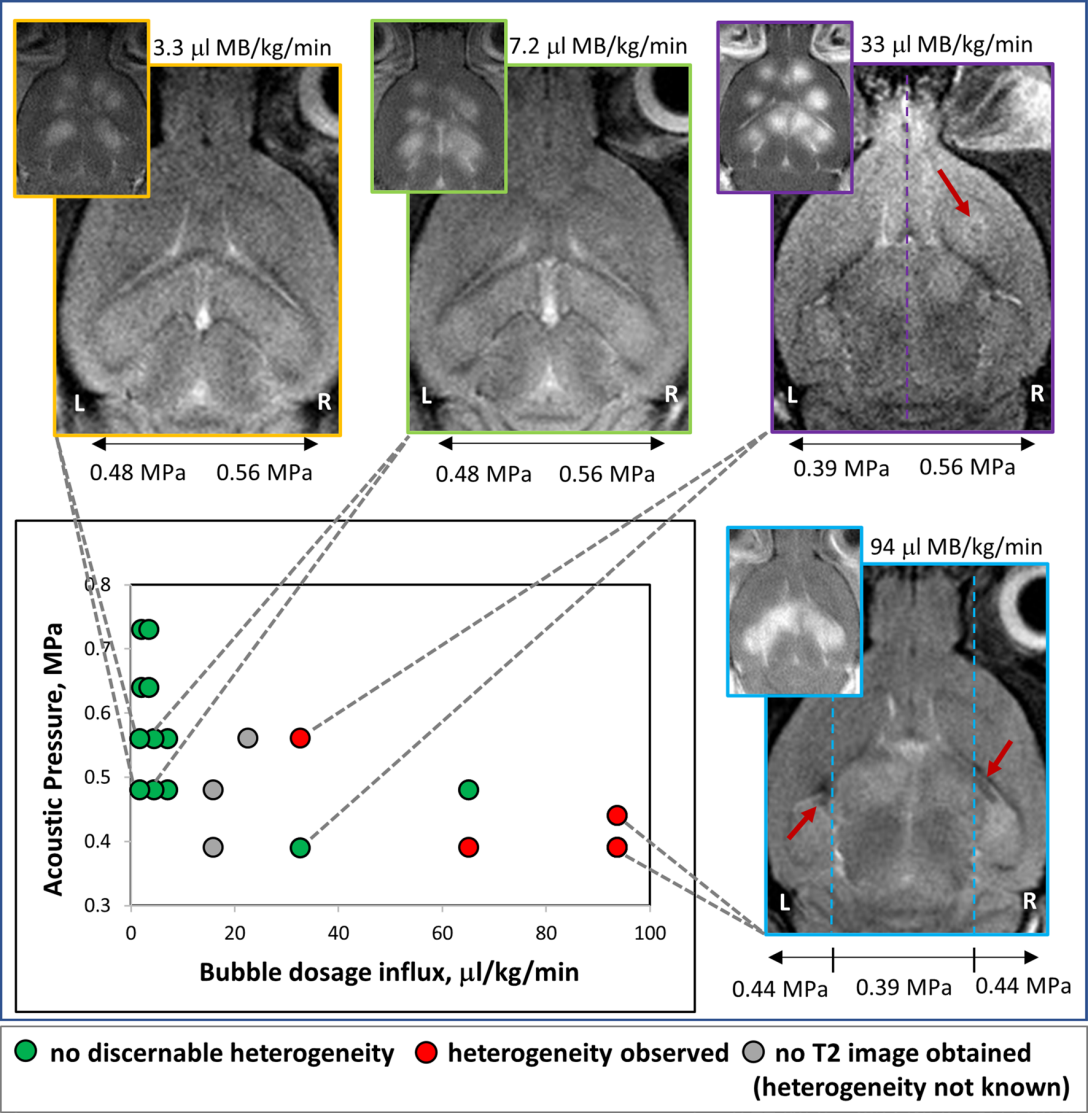
Representative images showing heterogeneity in brain parenchyma as indicated by T2-weighted MR images. T2-weighted images with corresponding inset of post-contrast T1-weighted images showing differences in T2 heterogeneity at some FUS treated locations. Case examples shown in images correspond to specific points on the plot of acoustic pressure versus bubble dosage influx (lower left, connected to images by dotted lines) where T2 heterogeneity was observed. WL and WW of images have been adjusted as stated in “Methods” section.

To assess the safety of our protocol at various bubble concentrations and acoustic pressures, T2-weighted MR images were obtained to assess for oedema or haemorrhage. Haemorrhage was primarily identified in cases with a higher bubble dosage rate (Fig. 6). When the data was further segregated by infusion rate, a higher microbubble infusion rate was more commonly associated with haemorrhage (83%, n = 6 animals) than a lower infusion rate (0%, n = 3 animals) was. Using a two-tailed two population proportion test for binary data, these results were significant at *p* = 0.0183 (see Supplementary Table S1 online).

## Discussion

The purpose of this study was to evaluate the efficacy of microbubble infusions for opening the BBB with FUS in multiple targets during a single treatment session. The effects of microbubble infusion rate and acoustic pressure were characterized, and compared to the standard using bolus injections.

In our infusion experiments, we observed consistent BBB opening across targets within individual animals for a broad range of microbubble dosage rates (Fig. 3). Over the course of the 6–10 min treatments, there was neither a substantial decay nor increase in enhancement rates. In contrast, our sample microbubble bolus experiments displayed wide variability in enhancement rates from point to point in single treatment sessions. Together with other studies that monitor microbubble activity through ultrasound imaging, harmonic emissions and BBB opening, these observations suggest that uniform microbubble concentrations are important in ensuring homogeneous BBB opening in multi-target treatments^22,28,30,31^. Due to the rapid rise and fall of intravenous microbubble concentration that occurs with bolus injections, variable enhancement rates are of concern in bolus delivery protocols. The use of infusions can potentially circumvent these problems and improve existing protocols that use bolus delivery.

Between individual animals there was substantial variation in enhancement rates (e.g. beyond a factor of two between animals dosed at 93.8 µl/kg/min, see Supplementary Table S1 online). We assume this was due to differences in skull insertion loss between animals, but it could also be inter-animal variability in the steady state bubble concentration during infusion.

Several groups have attempted to modify microbubble bolus protocols to improve the consistency of BBB opening during FUS treatment sessions with multiple targets. Wu et al. implemented an adaptive strategy of progressively increasing FUS exposure time for sequential points to compensate for relatively decreasing concentrations of microbubbles^31^. Chopra et al. introduced an interleaved sonication strategy where the ultrasound beam was rapidly scanned across all treatment points^35^. This approach approximated simultaneous sonication of all points at the same timepoints after injection of microbubbles. While these methods have demonstrated success, they are limited because they may be cumbersome to implement and impractical in the clinic or research laboratory.

Another group used microbubble harmonic emissions to modulate acoustic pressures for consistent BBB opening^22^. However, they found that bolus injections caused their feedback system to overcompensate. They found that an infusion of microbubbles even after bolus delivery allows for relatively uniform BBB opening.

An equally challenging problem is the overaccumulation of microbubbles in the blood. Such increased concentration after several injections can cause enhancement rates to elevate unintentionally. To avoid this issue, many groups have implemented delays between microbubble injections to allow for clearance^14,18,36^. Our results clearly show that even 15 min after delivery stopped there were enough circulating microbubbles for substantial BBB opening to occur (Fig. 5c,f). Therefore, care must be taken to avoid an inadvertent accumulation of microbubble concentration in the blood. Where multiple bolus doses per session may be necessary to treat larger regions of the brain, infusions eliminate this need and simplify the BBB opening protocol.

Acoustic pressure and microbubble influx rate are additional parameters that determine BBB opening. In our experiments, enhancement rates increased when microbubble concentrations increased up to influx rates of 32.7 µl/kg/min at 0.39 MPa, 15.9 µl/kg/min at 0.48 MPa and 22.7 µl/kg/min at 0.56 MPa. However, when bubble concentrations were increased further, enhancement rates unexpectedly decreased. This decrease was statistically significant at two of three acoustic pressures tested. Where significance was not found (Fig. 4b), it is likely that the peak enhancement rate had not been probed at this pressure. Other studies have also shown that after a certain concentration of microbubbles has been reached, the in vivo acoustic response to FUS plateaus and may drop off slightly^28,30^.

T2-weighted gradient echo magnetic resonance (MR) imaging has been used to detect oedema and/or haemorrhage after ultrasound exposure^29,37,38^. Past studies have examined the effect of acoustic pressure on FUS safety^26,39^, but effects of bubble dose on T2 changes have not been well characterized. Our observations suggest that higher bubble dose may contribute to injury (Fig. 6). Even at lower acoustic pressures, higher microbubble concentrations demonstrated more oedema and haemorrhage than when compared to experiments with lower microbubble concentrations and higher acoustic pressure.

Finally, in the infusion experiments, we observed inter-animal variability in enhancement rates. These findings could be related to differences in skull thickness which affect absorption of FUS energy. Additionally, variable microbubble clearance rates amongst animals could result in similar observations.

Limitations of this study existed in several areas. First, as T2 changes can be transient and resolve in repeat scans^29,40^, to assess the permanence of any tissue damage, we would have needed to acquire T2 images at later timepoints such as 6–24 h post treatment. Second, to acquire post-treatment contrast enhancement, we used a higher dose of contrast agent than used in most studies. This was done to ensure that enhancement rate was detectable even at the lowest microbubble doses; however, it may have affected the extent of apparent BBB opening. Third, we did not calibrate enhancement rates to any specific drug doses. Therefore, it was not possible for us to determine a lower recommended microbubble dosage limit at which a specific drug might be delivered at an effective dose.

As FUS mediated BBB opening moves into the early clinical phases, consistent, safe, and reliable BBB opening becomes a necessity. During bolus delivery, blood concentration of microbubbles varies greatly with time. Our work shows that continuous infusion during FUS treatment is able to maintain a sustained microbubble concentration in the blood, which allows for reliable robust BBB opening.

## Conclusions

This study demonstrates several benefits of microbubble infusions over bolus injections when targeting multiple sites for BBB disruption during a single FUS session. Infusions allow for more controlled, reproducible BBB opening across a variety of targets, and obviate complications from rapidly fluctuating microbubble concentrations. As such, infusions may play an important role in clinical treatments where multiple targets and larger volumes of BBB opening are required.

## Supplementary information

Supplementary Table S1.
