# Supplementary material for: Consistent opening of the blood brain barrier using focused ultrasound with constant intravenous infusion of microbubble agent
Source: Sci Rep. 2020 Oct 6;10:16546. doi: 10.1038/s41598-020-73312-9 (PMC7538995; doi:10.1038/s41598-020-73312-9)
Supplement: Supplementary file 1 — Supplementary Table S1. [file 41598_2020_73312_MOESM1_ESM.pdf]

Consistent Opening of the Blood Brain Barrier using Focused Ultrasound with Constant Intravenous Infusion of Microbubble Agent

Norman A. Lapin[1], Kirt Gill[1], Bhavya R. Shah[1,2,3,4,+] and Rajiv Chopra[1,4,*,+]

[1]Focused Ultrasound Laboratory, Department of Radiology, UT Southwestern Medical Center, Dallas, TX, 75390, USA
[2]Department of Neurosurgery, UT Southwestern Medical Center, Dallas, TX, 75390, USA
[3]Peter O'Donnell Jr. Brain Institute, UT Southwestern Medical Center, Dallas, TX, 75390, USA
[4]Advanced Imaging Research Center, UT Southwestern Medical Center, Dallas, TX, 75390, USA
*correspondence and requests for materials should be addressed to R.C.
Email: Rajiv.Chopra@utsouthwestern.edu
+co-senior authors

Total Dose Delivered* (µl bubble agent/kg)	Dosage influx/time (µl bubble agent/kg/min)	Acoustic pressure (MPa)	n treated points	ER mean	ER std dev	Parenchyma	n pts per parenchymal region	T2 heterogeneity observed?
**Delivery protocol: Constant infusion of microbubble agent**								
632	93.8	0.39	8	1.10	0.19	hippo/fctx	4/4	Y
641	93.8	0.39	8	1.05	0.14	hippo/fctx	4/4	Y
703	93.8	0.39	6	0.53	0.06	hippo	6	Y
		0.44	4	0.50	0.08	hippo (caud)	4	
703	93.8	0.39	6	1.22	0.10	hippo	6	Y
		0.44	4	1.33	0.07	hippo (caud)	4	
391	65.2	0.39	4	1.05	0.15	hippo/fctx	2/2	N
		0.48	4	1.17	0.08	hippo/fctx	2/2	
196	32.7	0.39	4	1.50	0.26	hippo/fctx	2/2	Y
		0.56	4	2.04	0.17	hippo/fctx	2/2	
136	22.7	0.56	8	2.44	0.28	hippo/fctx	4/4	not known
105	15.9	0.39	4	0.93	0.50	hippo/fctx	2/2	not known
		0.48	4	1.29	0.34	hippo/fctx	2/2	
42.9	7.2	0.48	4	0.87	0.13	hippo/fctx	2/2	N
		0.56	4	0.91	0.13	hippo/fctx	2/2	
27.0	4.5	0.48	4	0.50	0.21	hippo/fctx	2/2	N
		0.56	4	0.42	0.08	hippo/fctx	2/2	
10.4	1.7	0.48	4	0.26	0.08	hippo/fctx	2/2	N
		0.56	4	0.29	0.04	hippo/fctx	2/2	
**Microbubble agent bolus injections**								
87	3.3†	0.39	8	1.32	0.33	fctx	8	N
179	6.8†	0.39	8	0.76	0.32	fctx	8	Y

Supplementary Table S1. Summary of FUS treatment parameters, number of points and regions treated and resulting enhancement rates. Data provided for each animal treated include, from left to right: total bubble agent dosage delivered per animal treatment session, influx rate of bubble agent, acoustic pressure applied to each brain hemisphere during FUS, number of points treated at given FUS parameters, mean of the enhancement rates (ER) for all treated points in each treatment session, standard deviation of ERs of treated points, distribution of treated points broken down by

parenchymal region and number of points per region and whether T2 heterogeneity was observed post treatment. For infusions, total dose is calculated from time of bubble agent entry into mouse circulation through end of the last treated point included in the treatment session. This quantity does not account for removal of bubble agent by systemic clearance. *For infusions, total dose is calculated from time of bubble agent entry into mouse circulation through end of the last treated point included in the treatment session. This quantity does not account for removal of bubble agent by systemic clearance. †Dosage influx/time for bolus experiments is calculated as the total dose delivered (occurring within several seconds)/total FUS treatment time. hippo = hippocampus, caud = caudal, fctx = frontal cortex.
